# Reassortment of Influenza A Viruses in Wild Birds in Alaska before H5 Clade 2.3.4.4 Outbreaks

**DOI:** 10.3201/eid2304.161668

**Published:** 2017-04

**Authors:** Nichola J. Hill, Islam T.M. Hussein, Kimberly R. Davis, Eric J. Ma, Timothy J. Spivey, Andrew M. Ramey, Wendy Blay Puryear, Suman R. Das, Rebecca A. Halpin, Xudong Lin, Nadia B. Fedorova, David L. Suarez, Walter M. Boyce, Jonathan A. Runstadler

**Affiliations:** Massachusetts Institute of Technology, Cambridge, Massachusetts, USA (N.J. Hill, I.T.M. Hussein, K.R. Davis, E.J. Ma, W.B. Puryear, J.A. Runstadler);; University of Alaska Fairbanks, Alaska, USA (T.J. Spivey);; US Geological Survey, Anchorage, Alaska, USA (T.J. Spivey, A.M. Ramey);; Vanderbilt University Medical Center, Nashville, Tennessee, USA (S. Das);; J. Craig Venter Institute, Rockville, Maryland, USA (R.A. Halpin, X. Lin, N.B. Fedorova);; Department of Agriculture, Athens, Georgia, USA (D.L. Suarez);; University of California, Davis, California, USA (W.M. Boyce)

**Keywords:** influenza A virus, genetic reassortment, bird migration, spillover transmission, agricultural biosecurity, viruses, infectious diseases, mallards, ducks, poultry, wild birds, Alaska, H5 clade 2.3.4.4, avian, zoonoses

## Abstract

Sampling of mallards in Alaska during September 2014–April 2015 identified low pathogenic avian influenza A virus (subtypes H5N2 and H1N1) that shared ancestry with highly pathogenic reassortant H5N2 and H5N1 viruses. Molecular dating indicated reassortment soon after interhemispheric movement of H5N8 clade 2.3.4.4, suggesting genetic exchange in Alaska or surrounds before outbreaks.

The emergence of highly pathogenic avian influenza (HPAI) A virus subtype H5 of clade 2.3.4.4 in East Asia followed by spread into North America in 2014 highlights the importance of ecologic interactions along the Pacific Rim to the incursion of novel viruses. Introduction of influenza A subtype H5N8 into North America is hypothesized to have occurred through wild bird movement across the Bering Strait (*1*,*2*); the virus then spread through Canada to the continental United States, concurrently infecting wild birds (ducks, geese, passerines, and raptors) and poultry (turkeys and chickens) (*3*). Reassortment of H5N8 with low pathogenic avian influenza (LPAI) A virus in North America generated 3 subtypes (H5N8, H5N2, and H5N1, collectively referred to as H5Nx) that followed different trajectories in local bird populations. HPAI H5N2 became the most widespread in US poultry, prompting the culling of ≈49 million chickens and turkeys in 15 states (*4*). During the outbreaks (November 2014–December 2015), surveillance efforts increased; consequently, later stages of the epidemic were better characterized (*5*,*6*) relative to the beginning. Analysis of wild bird viruses from Alaska preceding outbreaks remains one of the few avenues for elucidating how H5N8 entered and reassorted with North American lineage viruses.

Our sampling of mallards (*Anas platyrhynchos*) from urban ponds in Anchorage, Alaska, during September 2014–April 2015 identified LPAI H5N2 and H1N1 (Technical Appendix Figure 1, Table 1 ). These viruses were the closest relatives for 4 of the 8 North American segments that contributed to the H5Nx reassortants based on the time of most recent common ancestry (tMRCA) analysis (Technical Appendix Figures 2–12). All North American segments of the H5N2 reassortant (basic polymerase protein 1, nucleoprotein, and neuraminidase) shared most recent common ancestry with LPAI H5N2 that circulated in Anchorage mallards and a concurrently sampled wild bird population at Izembek National Wildlife Refuge in western Alaska (*1*) (Technical Appendix Figures 3, 5, 8, 9). Molecular dating indicated reassortment of H5N8 and LPAI H5N2 (or precursors) shortly after the interhemispheric movement of H5N8. We estimated that ancestors of the H5N2 reassortant emerged among wild birds in Alaska during August 2013–May 2014, based on tMRCAs of multiple segments (Figure 1, panel A), followed by emergence of the H5N2 reassortant during May–September 2014. Our analysis refines the hypothesis of Beringia introduction (*1*,*2*) by indicating that H5N8 reassorted with viruses shed by waterfowl in Alaska (or nearby high latitudes) shortly after introduction into North America (May 2014–October 2014, 95% highest posterior density January 2014–January 2015) (Figure 1, panel A). Accuracy of molecular dating hinges on the availability of virus sequences from relevant hosts, which were lacking from North American poultry and wild birds during the year preceding outbreaks (Figure 1, panel B). Our sampling was fortuitous, being one of the few in Alaska conducted before the outbreaks, underscoring the importance of routine sampling where interhemispheric mixing of viruses occurs with high frequency.

**Figure 1:**
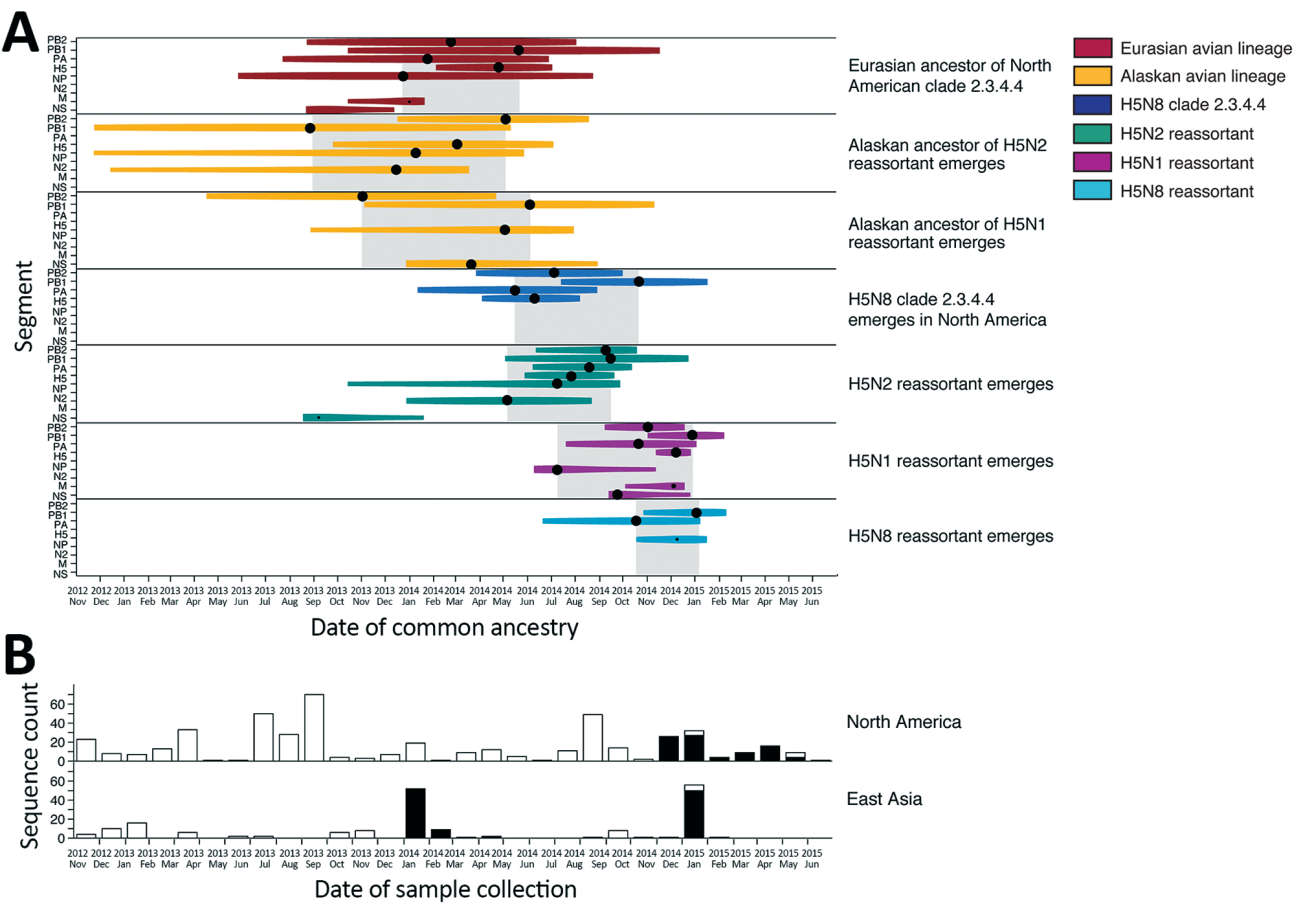
Molecular dating of the emergence of H5 clade 2.3.4.4 influenza A virus in Eurasia and North America and concurrent trends in surveillance effort. A) Events contributing to the evolution of H5 clade 2.3.4.4 estimated using multiple influenza segments. Time of most recent common ancestry (indicated by a black circle) is size-scaled by the posterior probability (0.0–1.0), and the 95% highest posterior density is color-coded by lineage. Gray shading indicates time of most recent common ancestry of multiple segments with a posterior probability >0.85. B) Surveillance effort estimated by the number of hemagglutinin sequences (high and low pathogenicity) available in the Influenza Research Database (https://www.fludb.org). Black bars indicate surveillance effort for H5 clade 2.3.4.4; white bars indicate surveillance effort for other clades. M, matrix gene; N2, neuraminidase 2 gene; NP, nucleoprotein gene; PA, polymerase acidic, PB1, basic polymerase protein 1 gene; PB2, basic polymerase protein 2 gene; tMRCA, time of most recent common ancestry.

The rapid reassortment of H5N8 clade 2.3.4.4 within North America is premised on the movement of at least a single infected bird across the Bering Strait followed by infection of a host population at high latitude. Interhemispheric movement during spring 2014 (or earlier) is most plausible given the circulation of the Eurasian ancestor of North American clade 2.3.4.4 during December 2013–May 2014 (95% highest posterior density October 2013–January 2015) (Figure 1, panel A), an event that preceded introduction. The presence of overwintering birds in Alaska, a known area for influenza exchange between East Asia and North America (*7*,*8*), might enhance opportunities for viruses originating in Eurasia to reassort with LPAI in local bird populations. Mallards from this study are a prime example of an overwintering population, occupying urban ponds that remained thawed because of human activity, which allows some birds to remain in southcentral Alaska from September through April, when many migratory waterfowl have since flown south. We found evidence that LPAI H5N2 shed by overwintering mallards from Anchorage (south-central Alaska) and wild birds from Izembek (western Alaska) were highly related and formed monophyletic clades (Technical Appendix Figures 5, 6, 8–10). This provided evidence of regional dispersal of LPAI in Alaska concurrent with the proposed timing of H5N8 introduction and reassortment.

Anchorage mallards shed viruses that shared ancestry with 2 of 4 North American segments (basic polymerase protein 1 and nonstructural) of the H5N1 reassortant (Technical Appendix Figures 2, 5, 12). Emergence of the H5N1 reassortant probably occurred after July 2014 (Figure 1, panel A), after the H5N2 reassortant emerged. Molecular dating of tMRCA of H5N8 reassortants was confounded by long branch lengths of parental lineages indicative of unsampled ancestors; however, estimates based on 2 segments suggest emergence after October 2014. The H5N1 and H5N8 reassortants possessed a highly similar polymerase acidic segment (Technical Appendix Figure 6), suggesting a similar evolutionary trajectory of the two subtypes that later diverged. Our tMRCA estimates for H5N1 are consistent with reassortment during or after the breeding season for mallards in Alaska followed by southward dispersal along the Pacific Flyway during autumn (Figure 2). These results suggest that H5N1 is a multiple reassortant that acquired PB1 and NS segments in Alaska (or surrounds) followed by polymerase acidic and neuraminidase 2 from different host populations before detection in Washington (*9*) and Oregon (*4*) in early 2015.

**Figure 2:**
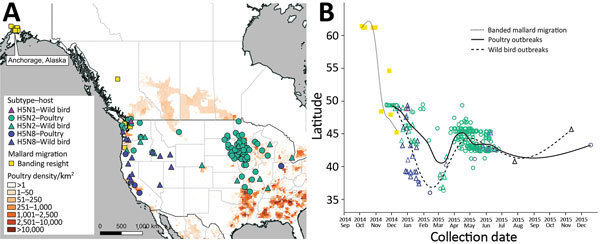
Spatial and temporal distribution of H5 clade 2.3.4.4 influenza A virus outbreaks among wild birds and poultry across North America. A) Spatial distribution of H5 clade 2.3.4.4 influenza A virus outbreaks in wild birds (triangles) and poultry (circles) across North America, color-coded by subtype, relative to poultry density. The location of mallards from Anchorage, Alaska, based on resighting of banded birds, is indicated. B) Temporal distribution of H5 clade 2.3.4.4 influenza A virus detections during the course of the outbreaks relative to the migration of mallards banded in Anchorage.

Arrival of mallards banded in Anchorage at the high-density poultry region of the Fraser Valley, British Columbia, Canada, in November 2014 is compatible with the chronology of evolution and subsequent detection of the H5Nx subtypes (Figure 2). The migration chronology of banded mallards might be broadly representative of other dabbling duck species that breed in Alaska, such as the American green-winged teal (*Anas carolinensis*), American wigeon (*Anas americana*), and northern pintail (*Anas acuta*), in which H5Nx was detected at lower latitudes. Consequently, mallards and other waterfowl species probably were involved in the southward dispersal and reassortment of H5Nx followed by spillover to poultry. Observations of wild birds congregating at water bodies on poultry farms in the Fraser Valley support the scenario of indirect transmission from migratory birds to poultry, seeding outbreaks at lower latitudes (*10*). Later divergence of H5N2 into multiple lineages during May–November 2014 (Technical Appendix Figures 8) implies that outbreaks were seeded by different H5N2 strains, although the mode of dispersal through wild bird migration, farm-to-farm poultry movement, or poultry workers remains unclear.

Since introduction of HPAI H5 of clade 2.3.4.4 into the Pacific Northwest in late 2014, little evidence exists for additional reassortment, despite continued spread of H5N2 and H5N8 until late 2015 (Figure 2, panel B). Lack of further reassortment implies a change from wild bird–mediated dispersal to intermittent spillover between wild birds and poultry or indirect transmission among poultry farms via fomites, wind, or other undetermined vectors. The spatiotemporal pattern of outbreaks in wild birds and poultry appeared correlated during this later phase (Figure 2, panel B). Correlation might be a function of outbreak investigation procedures that require concurrent sampling of poultry and wild birds inhabiting the control zone. However, our phylogenetic analysis lends support for frequent spillover given that lineages of H5Nx were mixed by host, rather than poultry and wild birds clustering separately (Technical Appendix Figures 4–12). Our analysis and the August 2016 detection of HPAI H5N2 in mallards from Fairbanks, Alaska (*11*), an area lacking commercial poultry, implicates waterfowl as playing an important role in reassortment, spread, and possibly long-term circulation of H5Nx viruses.

Technical Appendix Methods used for study of rapid reassortment of high and low pathogenic influenza A virus among wild birds in Alaska before H5 clade 2.3.4.4 outbreaks, including sampling of wild birds, banding data and outbreak analysis, virus isolation and sequencing, multiple sequence alignment, down-sampling before tree phylogenetic reconstruction, Bayesian phylogenetic analysis, and molecular dating analysis.
