# Supplementary material for: Reassortment of Influenza A Viruses in Wild Birds in Alaska before H5 Clade 2.3.4.4 Outbreaks
Source: Emerg Infect Dis. 2017 Apr;23(4):654–7. doi: 10.3201/eid2304.161668 (PMC5367406; doi:10.3201/eid2304.161668)
Supplement: Technical Appendix — Methods used for study of rapid reassortment of high and low pathogenic influenza A virus among wild birds in Alaska before H5 clade 2.3.4.4 outbreaks, including sampling of wild birds, banding data and outbreak analysis, virus isolation and sequencing, multiple sequence alignment, down-sampling before tree phylogenetic reconstruction, Bayesian phylogenetic analysis, and molecular dating analysis. [file 16-1668-Techapp-s1.pdf]

Article DOI: http://dx.doi.org/10.3201/eid2304.161668

Reassortment of Influenza A Viruses in Wild Birds in Alaska before H5 Clade 2.3.4.4 Outbreaks

Technical Appendix

Methods

Sampling of Wild Birds

The sampling locations were predominately at Cuddy Family Midtown Park, Anchorage, Alaska (61.184981°, –149.880597°) and Westchester Lagoon, Anchorage, Alaska (61.202333°, –149.90333°), two urban locations that attract overwintering mallards (online Technical Appendix Figure 1, panel A). Between September 2014 and April 2015 we sampled a total of 484 individual birds and recaptured 216. Baited swim and walk-in traps were used to capture mallards. We obtained a cloacal and tracheal swab from each bird and fitted each bird with an aluminum federal tarsus band. Polyester-tipped applicators were used to collect swabs, placed in viral transport media (VTM: Remel, KS) and kept on ice until transfer to –80 freezers in the laboratory.

Banding Data and Outbreak Analysis

We obtained recovery/recapture data for mallards banded in Anchorage from the U.S. Geological Survey Bird Banding Laboratory. Of the 468 ducks banded, a total of 75 (16.03%) bands were reported between 2014 and 2016, mostly representing hunter-shot ducks. Band returns were mapped using QGIS 2.0 (Open Source Geospatial Foundation Project. http://qgis.osgeo.org) to describe connectivity of the Anchorage mallard population with other regions in North America. Poultry (chicken) density layers were obtained from the Gridded Livestock of the World (*1*) available online. Location and timing of all H5 clade 2.3.4.4 outbreaks were obtained from federal agency Web sites for the U.S. (U.S. Department of Agriculture [*2*]) and Canada (Canadian Food Inspection Agency [*3*]). A smoothing average for the spatiotemporal pattern of outbreaks was plotted separately for wild bird and poultry cases

separately (JMP Pro 12.2, SAS), as well as the chronology of mallard migration (Figure 2, panel B).

Virus Isolation and Sequencing

Viral RNA was extracted using the Omega Mag-Bind Viral DNA/RNA Kit (Omega Bio-Tek, Norcross, GA) and a Kingfisher magnetic particle processor (Thermo Scientific, Waltham, MA). RNA was screened for the presence of influenza A virus (IAV) using qScript XLT one-step RT-qPCR Tough Mix (Quanta Biosciences, Gathersburg, MD) targeting the matrix gene. PCR assays were run on an ABI 7500 real-time PCR System (Applied Biosystems, Foster City, CA). PCR-positive samples (Ct value <45) were subjected to a H5-specific rRT-PCR to identify potentially highly pathogenic samples (*4*). All H5-positive samples were sent to the U.S. National Poultry Research Center, USDA to culture potential HPAI viruses. To amplify virus, positive VTM (100ul) was inoculated into the allanotic cavity of 9–11 day old embryonating specific pathogen free chicken eggs (Charles River, CT) and incubated at 37°C for 72 hours or until embryo death, as detected by daily candling. RNA was extracted from the allantoic fluid and the matrix rRT-PCR repeated (*5*). Whole-genome sequencing was performed at the J. Craig Venter Institute in Rockville, MD as described by Nelson et al. (*6*) and all sequences deposited into GenBank (online Technical Appendix Table 1). Subtyping was confirmed by BLASTn of the sequence against isolates in GenBank and identifying the subtype match that showed highest percentage identity.

Multiple Sequence Alignment

Global IAV sequences were downloaded from the Influenza Research Database (IRD [*7*]) and Global Initiative on Sharing All Influenza Data (GISAID) during the period between 1/17/16 - 2/16/16. Only full genomic segments (PB2, PB1, PA, H5, NP, N2, N1, M and NS) of isolates collected during the period (1976 – 2016) were considered. Only sequences associated with a full date of collection (DD/MM/YYYY) were included. To facilitate filtering out duplicate sequences, taxa names were edited to remove apostrophes, brackets and all hyphens were replaced with underscores. Sequences containing misreads (NNNs) were deleted to improve the quality of alignments and subsequent trees. Sequences were then aligned using MUSCLE v3.8.31 (*8*), inspected visually in Geneious v7.1.5 to remove indels and taxa containing premature termination codons, then re-aligned once again using MUSCLE.

Down-Sampling before Tree Reconstruction

The aligned global sequence datasets were down-sampled to reduce the number of sequences to a size suitable for molecular clock Bayesian phylogenetic analysis (~300 taxa). The goal of down-sampling was 3-fold: 1) preserve all clade 2.3.4.4 isolates from North America; 2) preserve basal avian and mammalian lineages to accurately root the tree in deep evolutionary time; and 3) random down-sampling of non-clade 2.3.4.4 isolates stratified by time (3–5 isolates randomly preserved per year) to ensure confidence in tree topology from root to tips (online Technical Appendix Figure 13). Down-sampling was conducted using a series of Python scripts (available for download: 10.5281/zenodo.61923). A preliminary phylogenetic tree using the global sequence alignment for each segment was constructed using the GTRGAMMA substitution model in RAxML v 8.1.16 (*9*). The resulting maximum likelihood trees were used to compute patristic distances between a designated 'taxa-of-interest' group and the rest of the global dataset. This taxa-of-interest group included recent isolates from Anchorage and Izembek (2014) and all clade 2.3.4.4 isolates from North America.

To determine the minimum number of closest relatives to the taxa-of-interest needed to construct a well-sampled Bayesian phylogenetic tree we evaluated 3 metrics in TreeStat v1.8.2: i) time of most recent common ancestry (tMRCA); ii) tree height/root age; and iii) treeness (tree shape). These values were generated for the closest 10 and 25 relatives of the in-group by constructing preliminary Bayesian trees in BEAST v 1.8.3 (*10*). At least 3 independent Bayesian Markov Chain Monte Carlo (MCMC) chains, of 40 – 80 million generations each, were run and sampled under the Bayesian skyride coalescent model and the uncorrelated lognormal distribution, to produce 10,000 trees. In all cases we used a GTR + gamma substitution model. MCMC chains were visually assessed in Tracer v1.6.0 (http://tree.bio.ed.ac.uk/software/tracer) to ensure convergence on the same optimal tree. After removing 10 – 30% burn-in from each chain we combined trees and constructed maximum clade credibility trees. High similarity between the closest 10 and 25 relatives for the 3 metrics tested (online Technical Appendix Table 2) validated our decision to use the closest 10 relatives to reduce computational burden without compromising the reproducibility of the Bayesian trees. This data-driven approach guided how to down-sample the internal segments. For the H5 and N2 segments, we included the closest 100 relatives, and for N1, we included the closest 25 relatives owing to the limited taxa available.

To achieve down-sampling stratified by time – a strategy we used to increase accuracy of molecular dating of divergence events, a maximum of 100 taxa were randomly selected from our global datasets, spanning 1996–2016, with an average of 3–5 isolates per year. The 1996 cutoff was chosen because it marks the emergence of the A/goose/Guangdong/1/1996 lineage. A compiled BEAST dataset was obtained by combining recent Alaskan isolates, the in-group of clade 2.3.4.4 isolates and randomly selected taxa. The down-sampled datasets contained the following numbers of taxa: PB2 = 321, PB1 = 334, PA = 309, H5 = 370, NP = 347, N2 = 311, N1 = 277, M = 362, and NS = 374.

Bayesian Phylogenetic Analysis

Dated phylogenetic trees for each segment were constructed using BEAST v1.8.3. At least 4 independent MCMC chains, of 40 – 80 million generations each, were run using the GMRF Bayesian skyride coalescent tree prior and uncorrelated lognormal distribution to produce 10,000 trees. To ensure MCMC chains converged on the same optimal tree, runs were visually inspected in Tracer v1.6.0. After removing 10 – 30% burn-in from each chain, the trees were combined to produce a single maximum clade credibility (MCC) tree.

Molecular Dating Analysis

The time of most recent common ancestry (tMRCA) for each taxon was inferred from the nodes of the MCC tree inspected in FigTree v1.4.3. The tMRCA dates and 95% highest posterior densities (95% HPD) for all segments were used to assess the order of gene introduction events (Figure 1, panel A). We also considered the posterior probabilities associated with each branch node when estimating gene segment introduction events. Only gene segment introductions associated with a posterior probability greater than 0.85 were considered such that bifurcating nodes with low support were ignored. We considered this an important step in view of the uneven sampling regime (targeting poultry farms) and low number of viruses sequenced during the outbreak period. As a result, not all influenza segments contributed equally to this analysis. For the matrix (M) and non-structural (NS) genes, low posterior probabilities for branch nodes (Technical Appendix Figure 12) combined with reverse branching (ambiguity in the exact timing of divergence events) precluded their use for estimating gene introduction events. These conserved segments were of limited value for estimating the rapid evolution of H5 clade 2.3.4.4, especially in view of the low number of sequences available before the outbreaks.

References

1. Robinson TP, Wint GR, Conchedda G, Van Boeckel TP, Ercoli V, Palamara E, et al. Mapping the global distribution of livestock. PLoS One. 2014;9:e96084. PubMed http://dx.doi.org/10.1371/journal.pone.0096084

2. US Department of Agriculture. Avian influenza disease. Animal disease information. Avian health [cited 2016 Aug 5]. https://www.aphis.usda.gov/aphis/ourfocus/animalhealth/animal-disease-information/avian-influenza-disease

3. Canadian Food Inspection Agency. Avian Influenza - British Columbia Infected Premises. Reportable diseases: avian influenza [cited 2016 August 5]. http://www.inspection.gc.ca/animals/terrestrial-animals/diseases/reportable/ai/2014-2015-ai-investigation-in-bc/infected-premises/eng/1418340527324/1418340584180

4. Wang R, Soll L, Dugan V, Runstadler J, Happ G, Slemons RD, et al. Examining the hemagglutinin subtype diversity among wild duck-origin influenza A viruses using ethanol-fixed cloacal swabs and a novel RT-PCR method. Virology. 2008;375:182–9. PubMed http://dx.doi.org/10.1016/j.virol.2008.01.041

5. Eisfeld AJ, Neumann G, Kawaoka Y. Influenza A virus isolation, culture and identification. Nat Protoc. 2014;9:2663–81. PubMed http://dx.doi.org/10.1038/nprot.2014.180

6. Nelson MI, Simonsen L, Viboud C, Miller MA, Holmes EC. Phylogenetic analysis reveals the global migration of seasonal influenza A viruses. PLoS Pathog. 2007;3:1220–8. PubMed http://dx.doi.org/10.1371/journal.ppat.0030131

7. Squires RB, Noronha J, Hunt V, García-Sastre A, Macken C, Baumgarth N, et al. Influenza research database: an integrated bioinformatics resource for influenza research and surveillance. Influenza Other Respi Viruses. 2012;6:404–16. PubMed http://dx.doi.org/10.1111/j.1750-2659.2011.00331.x

8. Edgar RC. MUSCLE: multiple sequence alignment with high accuracy and high throughput. Nucleic Acids Res. 2004;32:1792–7. PubMed http://dx.doi.org/10.1093/nar/gkh340

9. Stamatakis A. RAxML-VI-HPC: maximum likelihood-based phylogenetic analyses with thousands of taxa and mixed models. Bioinformatics. 2006;22:2688–90. PubMed http://dx.doi.org/10.1093/bioinformatics/btl446

10. Drummond AJ, Suchard MA, Xie D, Rambaut A. Bayesian phylogenetics with BEAUti and the BEAST 1.7. Mol Biol Evol. 2012;29:1969–73. PubMed http://dx.doi.org/10.1093/molbev/mss075

Technical Appendix Table 1. List of 12 influenza A virus strains (H5N2 and H1N1) isolated from mallards in Anchorage, Alaska, that were precursors to the reassortment event with highly pathogenic H5N8 clade 2.3.4.4

Strain name	Accession numbers	Subtype	Sampling date	Swab type	Mallard age	Band number
A/mallard/Southcentral Alaska/12ML00957/2014	CY206693-CY206700	H1N1	19-Sep-14	Cloacal	Hatch-year	2047–55491
A/mallard/Southcentral Alaska/12ML00991/2014	CY194156-CY194163	H5N2	22-Sep-14	Cloacal	Hatch-year	2047–55121
A/mallard/Southcentral Alaska/12ML00985/2014	CY194147-CY194155	H5/3N2	22-Sep-14	Cloacal	Hatch-year	2047–55110
A/mallard/Southcentral Alaska/12ML00988_AAF1/2014	CY194181-CY194188	H5N2	22-Sep-14	Oral	Hatch-year	2047–55104
A/mallard/Southcentral Alaska/12ML00977/2014	CY194139-CY194146	H5N2	22-Sep-14	Cloacal	Hatch-year	2047–55107
A/mallard/Southcentral Alaska/12ML00995/2014	CY206733-CY206740	H1N1	22-Sep-14	Cloacal	Hatch-year	2047–55113
A/mallard/Southcentral Alaska/12ML01009/2014	CY206741-CY206748	H1N1	22-Sep-14	Cloacal	Hatch-year	2047–55117
A/mallard/Southcentral Alaska/12ML01048_AAF1/2014	CY194213-CY194220	H5N2	23-Sep-14	Cloacal	Hatch-year	2047–55138
A/mallard/Southcentral Alaska/12ML01045/2014	CY206781-CY206788	H1N1	23-Sep-14	Cloacal	Hatch-year	2047–55136
A/mallard/Southcentral Alaska/12ML01175/2014	CY206797-CY206805	H1N1	20-Oct-14	Cloacal	After hatch-year	2047–55005
A/mallard/Southcentral Alaska/12ML01443/2014	CY206806-CY206813	H1N1	24-Oct-14	Cloacal	Hatch-year	2047–55025
A/mallard/Southcentral Alaska/12ML01469/2014	CY194164-CY194172	H5/9N2	24-Oct-14	Cloacal	Hatch-year	2047–55065

Technical Appendix Table 2. Time of most recent common ancestry (tMRCA) for each of the 8 influenza segments

Segment	Emergence event	Posterior probability	tMRCA	Maximum tMRCA	Minimum tMRCA
PB2	Eurasian ancestor of North American clade 2.3.4.4	**0.999**	23-Feb-14	27-Jul-14	6-Sep-13
PB2	H5N8 clade 2.3.4.4 emerges in North America	**1.000**	5-Jul-14	27-Sep-14	4-Apr-14
PB2	H5N1 reassortant emerges	**0.881**	2-Nov-14	17-Dec-14	12-Sep-14
PB2	H5N2 reassortant emerges	**0.960**	9-Sep-14	19-Oct-14	15-Jun-14
PB2	Alaskan ancestor of H5N2 reassortant	**1.000**	4-May-14	14-Aug-14	27-Dec-13
PB2	Alaskan ancestor of H5N1 reassortant	**1.000**	2-Nov-13	15-Apr-14	2-May-13
PB1	Eurasian ancestor of North American clade 2.3.4.4	**1.000**	12-May-14	2-Nov-14	21-Oct-13
PB1	H5N8 clade 2.3.4.4 emerges in North America	**1.000**	22-Oct-14	8-Jan-15	9-Jul-14
PB1	Alaskan ancestor of H5N2 reassortant	**0.999**	22-Aug-13	16-Apr-14	1-Dec-12
PB1	H5N2 reassortant emerges	**1.000**	15-Sep-14	19-Dec-14	13-May-14
PB1	Alaskan ancestor of reassortant H5N1	**1.000**	31-May-14	30-Oct-14	16-Nov-13
PB1	H5N1 reassortant emerges	**1.000**	25-Dec-14	1-Feb-15	23-Oct-14
PB1	H5N8 reassortant emerges	**1.000**	28-Dec-14	9-Feb-15	6-Nov-14
PA	Eurasian ancestor of North American clade 2.3.4.4	**1.000**	24-Jan-14	22-Jun-14	6-Aug-13
PA	H5N8 clade 2.3.4.4 emerges in North America	**1.000**	16-May-14	25-Aug-14	21-Jan-14
PA	North American ancestor of H5N8 & H5N1 reassortant	**1.000**	10-Mar-14	7-Nov-14	7-May-13
PA	H5N2 reassortant emerges	**1.000**	19-Aug-14	10-Oct-14	13-Jun-14
PA	H5N8 reassortant emerges	**1.000**	18-Oct-14	4-Jan-15	29-Jun-14
PA	H5N1 reassortant emerges	**0.995**	21-Oct-14	31-Dec-14	27-Jul-14
H5	Eurasian ancestor of North American clade 2.3.4.4	**1.000**	25-Apr-14	30-Jun-14	10-Feb-14
H5	H5N8 clade 2.3.4.4 emerges in North America	**0.999**	10-Jun-14	5-Aug-14	9-Apr-14
H5	North American ancestor of H5N8 & H5N1 reassortant	**0.992**	1-Nov-14	20-Nov-14	27-Sep-14
H5	H5N1 reassortant emerges	**1.000**	8-Dec-14	26-Dec-14	14-Nov-14
H5	H5N2 reassortant emerges	**0.998**	27-Jul-14	18-Sep-14	2-Jun-14
H5	Alaskan ancestor of H5N2 reassortant	**1.000**	3-Mar-14	29-Jun-14	7-Oct-13
H5	H5N8 reassortant emerges	0.225	9-Dec-14	11-Jan-15	15-Oct-14
NP	Eurasian ancestor of North American clade 2.3.4.4	**0.990**	24-Dec-13	15-Aug-14	15-Jun-13
NP	H5N2 reassortant emerges	**0.993**	9-Jul-14	24-Sep-14	26-Oct-13
NP	Alaskan ancestor of H5N2 reassortant	**0.992**	9-Jan-14	23-May-14	11-Dec-12

Segment	Emergence event	Posterior probability	tMRCA	Maximum tMRCA	Minimum tMRCA
NP	Alaskan ancestor of H5N1 reassortant	**0.806**	3-May-14	28-Jul-14	7-Sep-13
N1	H5N1 reassortant emerges	**1.000**	9-Jul-14	9-Nov-14	16-Jun-14
N2	H5N2 reassortant emerges	**0.992**	6-May-14	18-Aug-14	7-Jan-14
N2	Alaskan ancestor of H5N2 reassortant	**0.918**	15-Dec-13	12-Mar-14	1-Jan-13
M	Eurasian ancestor of North American clade 2.3.4.4	0.005	1-Jan-14	19-Jan-14	18-Oct-13
NS	Eurasian ancestor of North American clade 2.3.4.4	0.001	24-Sep-13	12-Dec-13	25-Aug-13
NS	Alaskan ancestor of reassortant H5N1	**0.996**	21-Mar-14	29-Aug-14	3-Jan-14
NS	H5N1 reassortant emerges	**0.999**	24-Sep-14	26-Dec-14	16-Sep-14
NS	H5N2 reassortant emerges	0.001	7-Sep-13	19-Jan-14	22-Aug-13

*tMRCA estimates with posterior probabilities >0.85 are highlighted in bold. Segments with Alaskan ancestry based on phylogenetic trees (online Technical Appendix Figures 4–12) are shaded in gray.

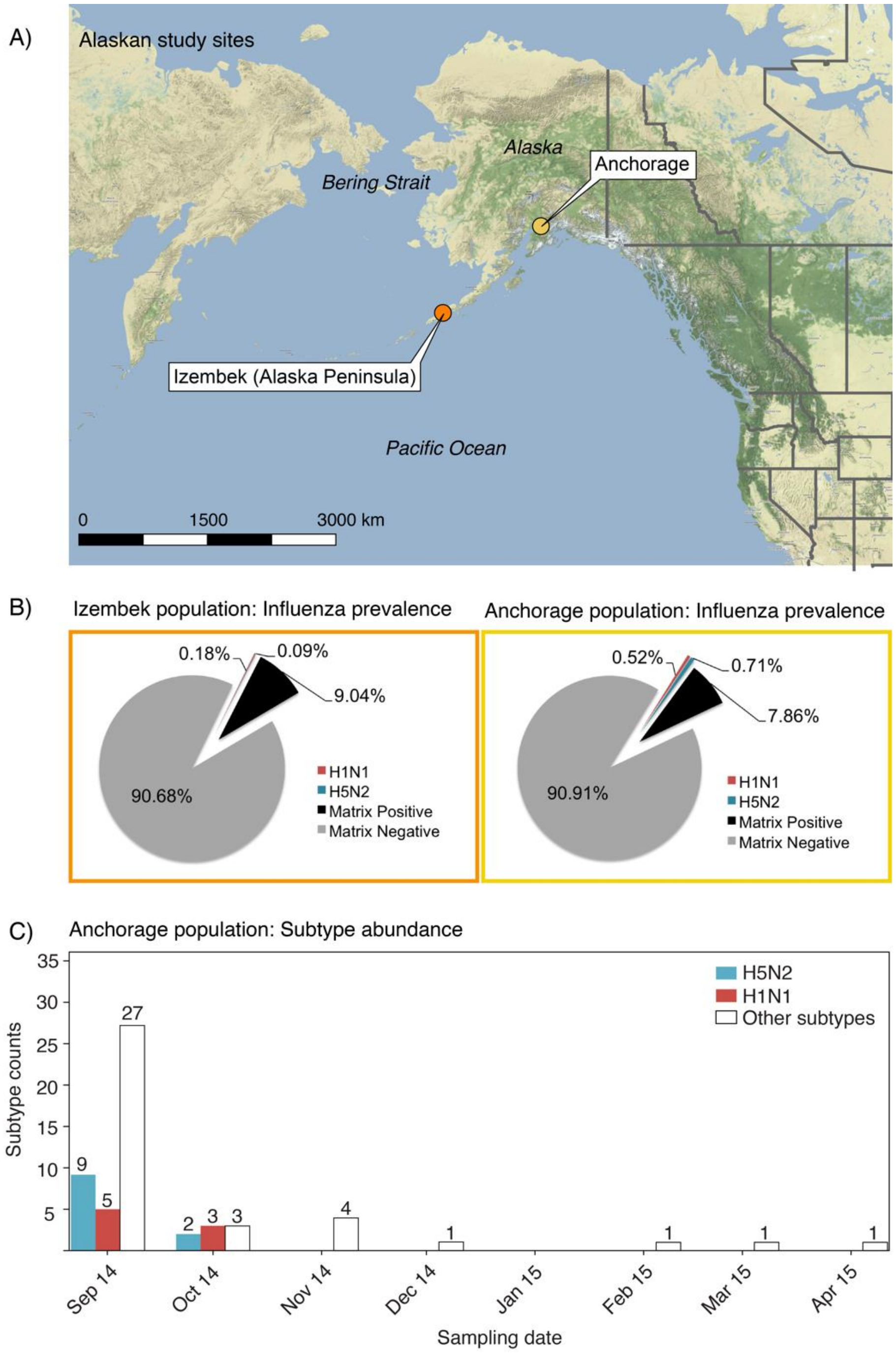

Technical Appendix Figure 1. Location of the Anchorage study site relative to the concurrently sampled Izembek site and prevalence of influenza A virus and the low pathogenic H5N2 subtype at each of the 2 sites. A) Location of the Anchorage study site relative to the concurrently sampled Izembek site, Alaska Peninsula. These were the only sites sampled in Alaska before the 2014 outbreaks. B) Prevalence of influenza A virus and the low pathogenic H5N2 subtype at each of the 2 sites, 2014–2015. C) Abundance of the low pathogenic H5N2 and H1N1 subtypes over the course of the winter among the Anchorage mallard population.

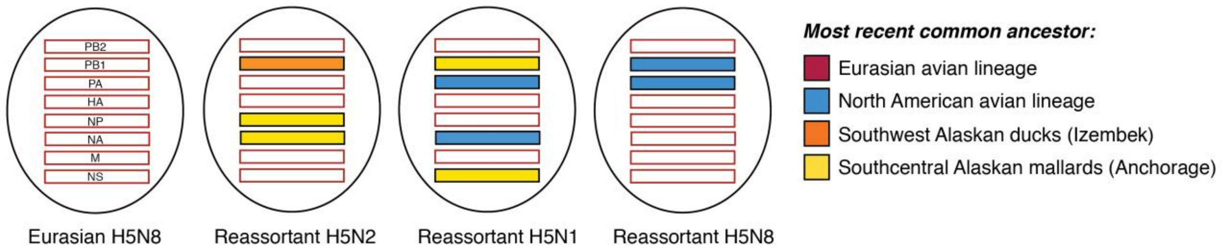

Technical Appendix Figure 2. Proposed genotypes of highly pathogenic influenza A virus H5Nx (reassortant H5N2, H5N1 and H5N8). Segments are color coded according to the most recent common ancestor for each genotype, based on phylogenetic tree reconstructions (Technical Appendix Figures 4–13).

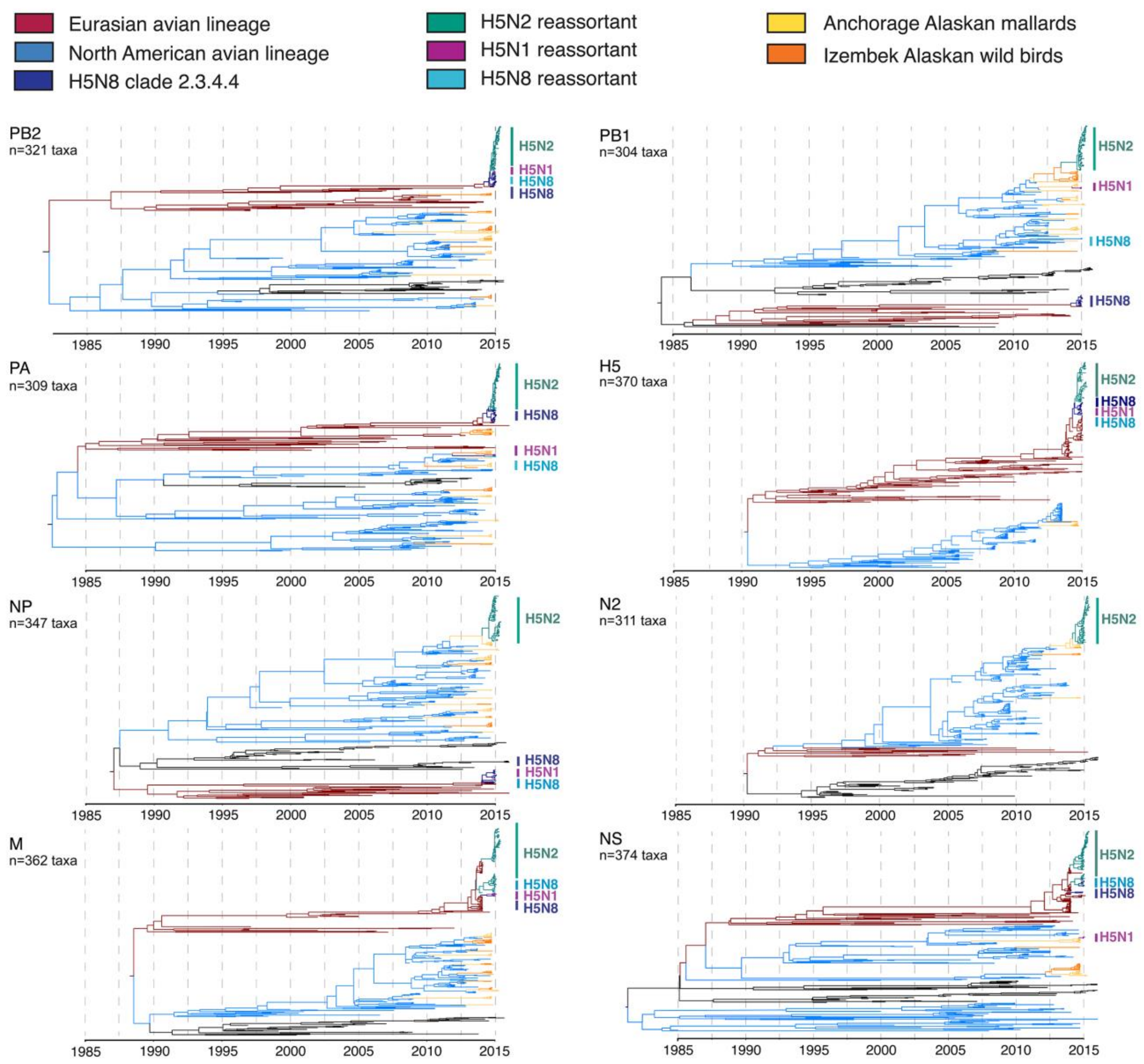

Technical Appendix Figure 3. Phylogenetic tree reconstruction using Bayesian inference was used to date the ancestry of clade 2.3.4.4 viruses for each of the 8 influenza segments. The ancestry of the 3 subtypes: H5N8 (blue), H5N2 (green), H5N1 (purple) is shown relative to viruses shed by the Alaskan wild populations from Anchorage (yellow) and Izembek (orange).

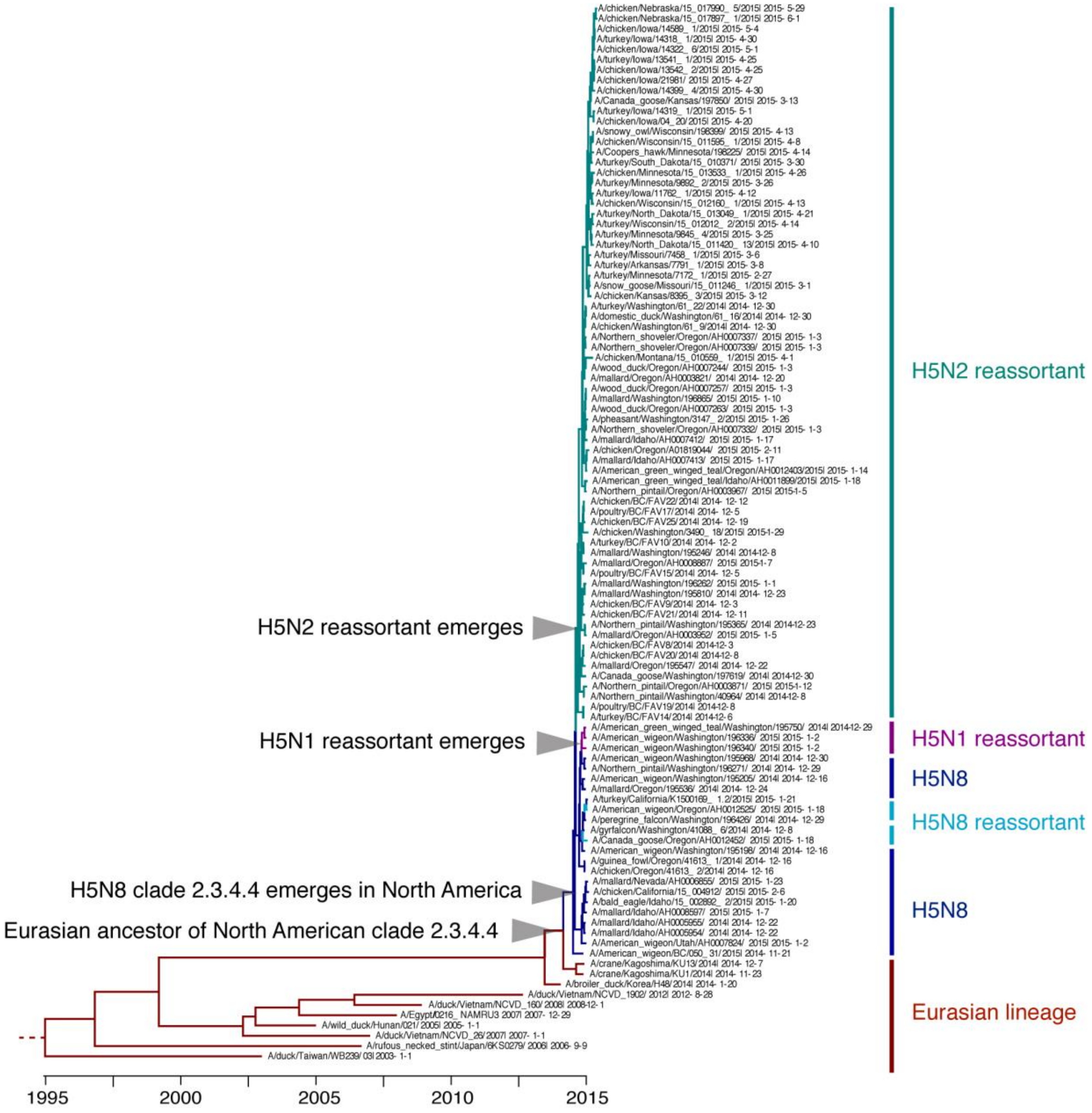

Technical Appendix Figure 4. Bayesian phylogenetic tree for the PB2 segment constructed using sequences from clade 2.3.4.4 North American outbreaks. The tree has been time-calibrated using a relaxed molecular clock and branch lengths represent years. Nodes used to calculate time of most recent common ancestry (tMRCA: posterior probabilities >0.85) are marked with gray arrows.

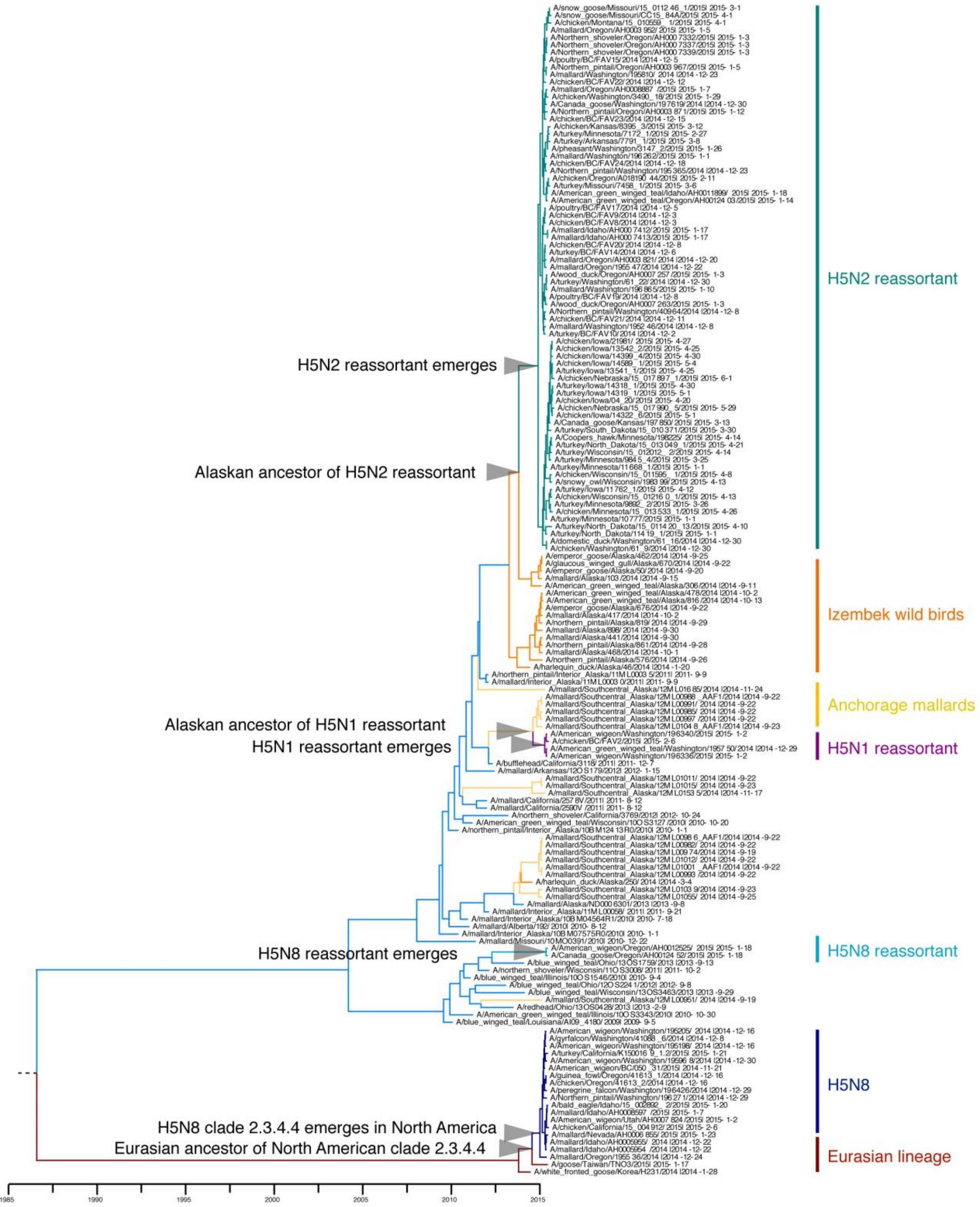

Technical Appendix Figure 5. Bayesian phylogenetic tree for the PB1 segment constructed using sequences from clade 2.3.4.4 North American outbreaks. The tree has been time-calibrated using a relaxed molecular clock and branch lengths represent years. Nodes used to calculate time of most recent common ancestry (tMRCA: posterior probabilities >0.85) are marked with gray arrows.

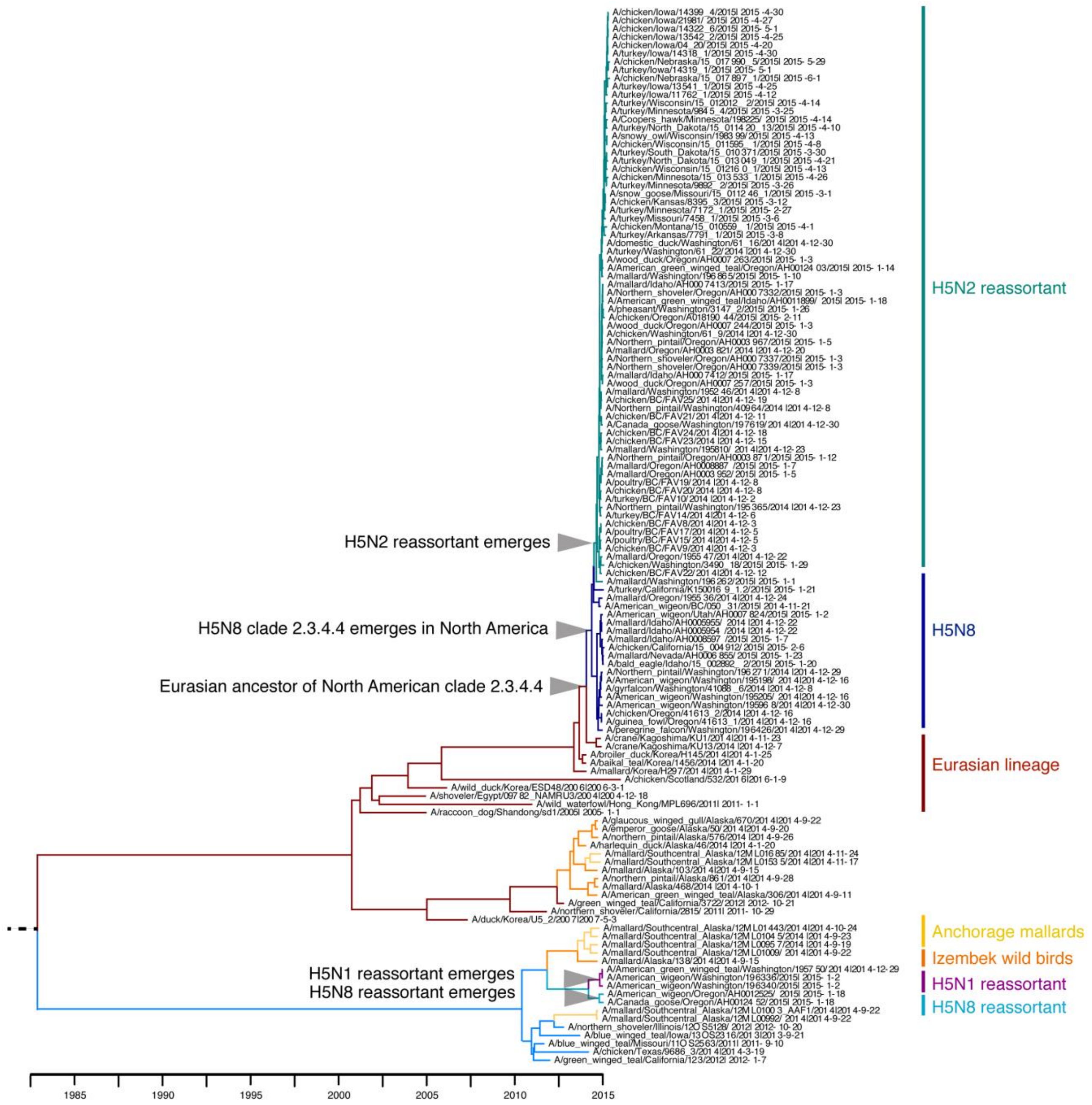

Technical Appendix Figure 6. Bayesian phylogenetic tree for the PA segment constructed using sequences from clade 2.3.4.4 North American outbreaks. The tree has been time-calibrated using a relaxed molecular clock and branch lengths represent years. Nodes used to calculate time of most recent common ancestry (tMRCA: posterior probabilities >0.85) are marked with gray arrows.

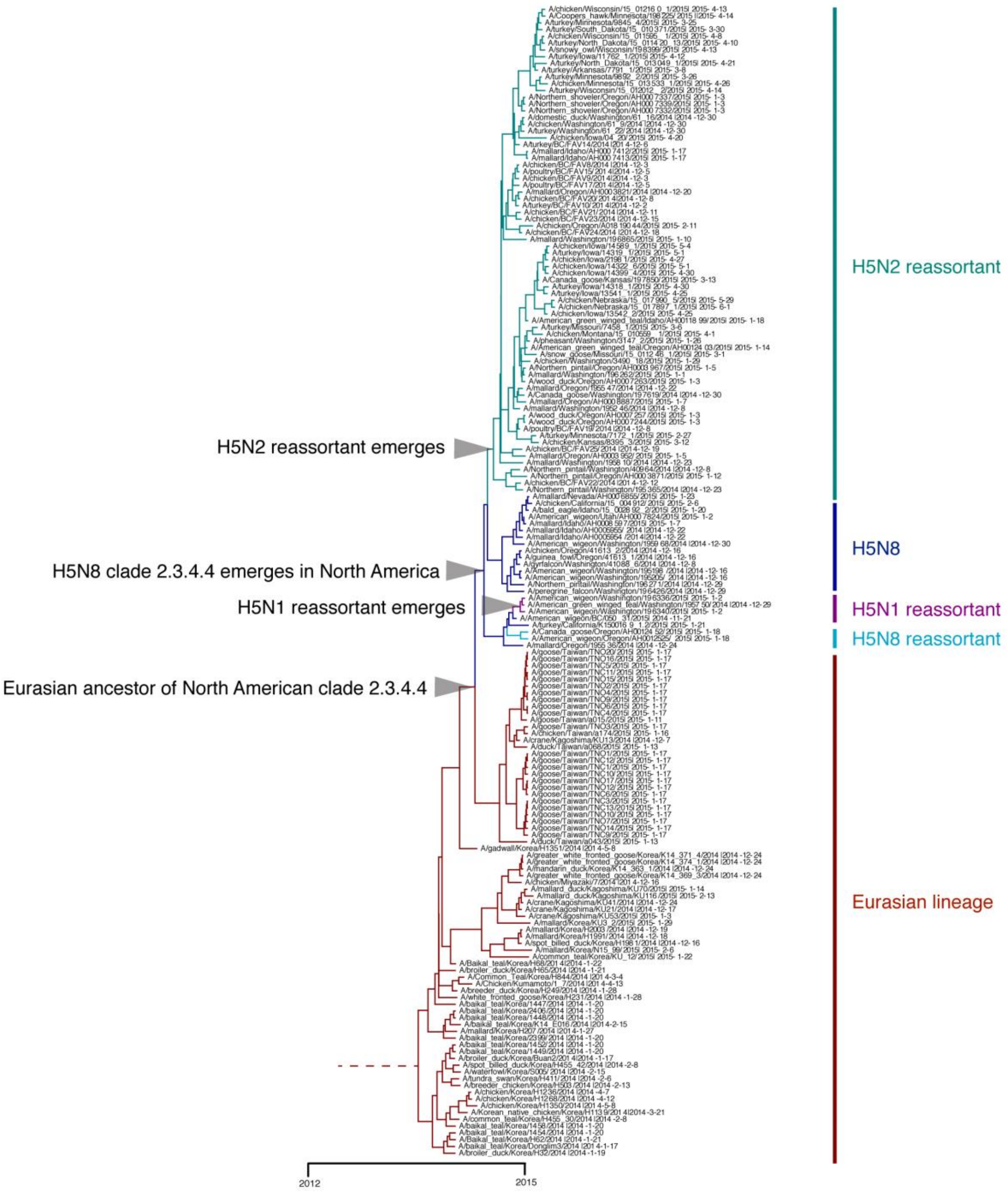

Technical Appendix Figure 7. Bayesian phylogenetic tree for the H5 segment constructed using sequences from clade 2.3.4.4 North American outbreaks. The tree has been time-calibrated using a relaxed molecular clock and branch lengths represent years. Nodes used to calculate time of most recent common ancestry (tMRCA: posterior probabilities >0.85) are marked with gray arrows.

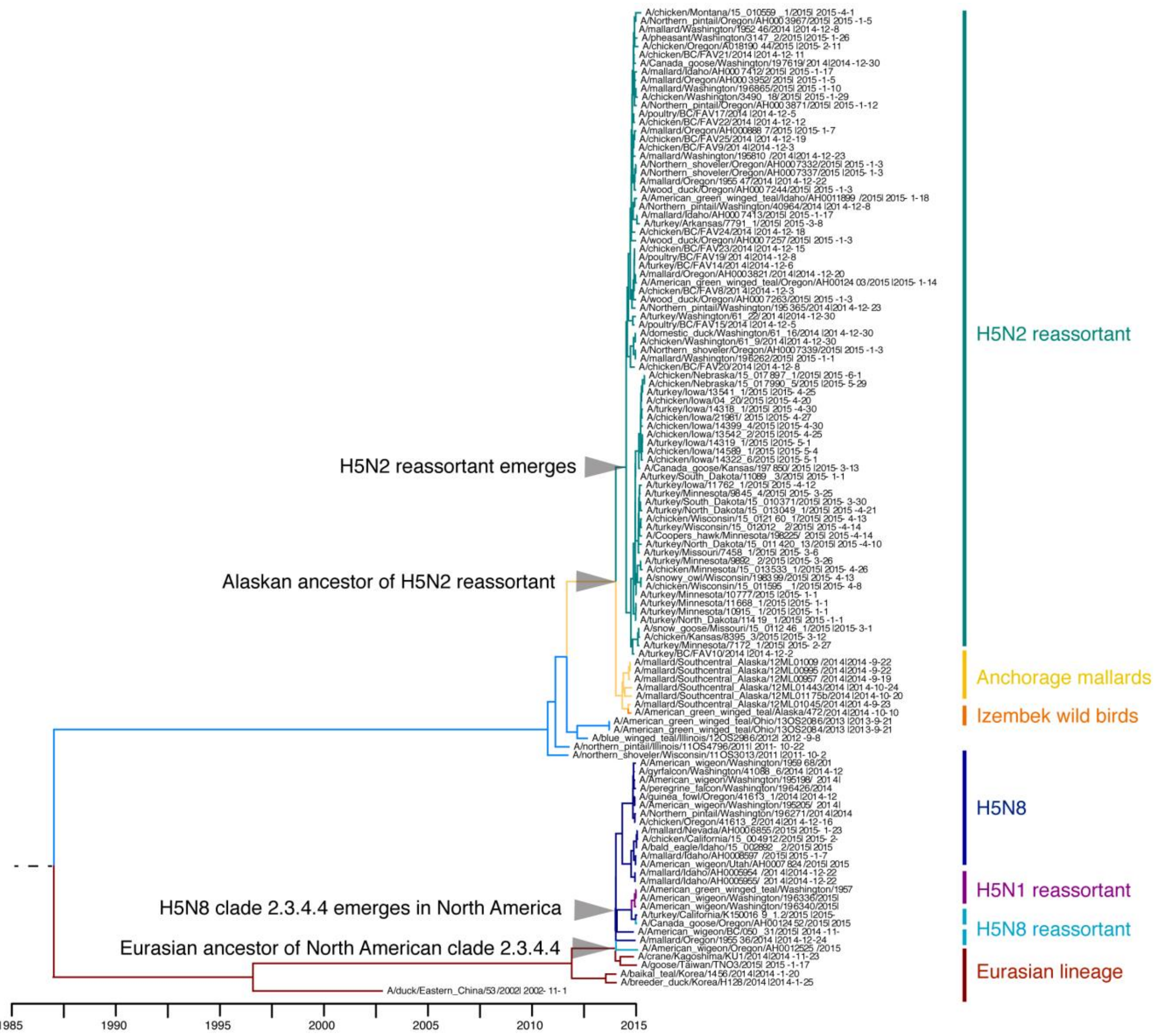

Technical Appendix Figure 8. Bayesian phylogenetic tree for the NP segment constructed using sequences from clade 2.3.4.4 North American outbreaks. The tree has been time-calibrated using a relaxed molecular clock and branch lengths represent years. Nodes used to calculate time of most recent common ancestry (tMRCA: posterior probabilities >0.85) are marked with gray arrows.

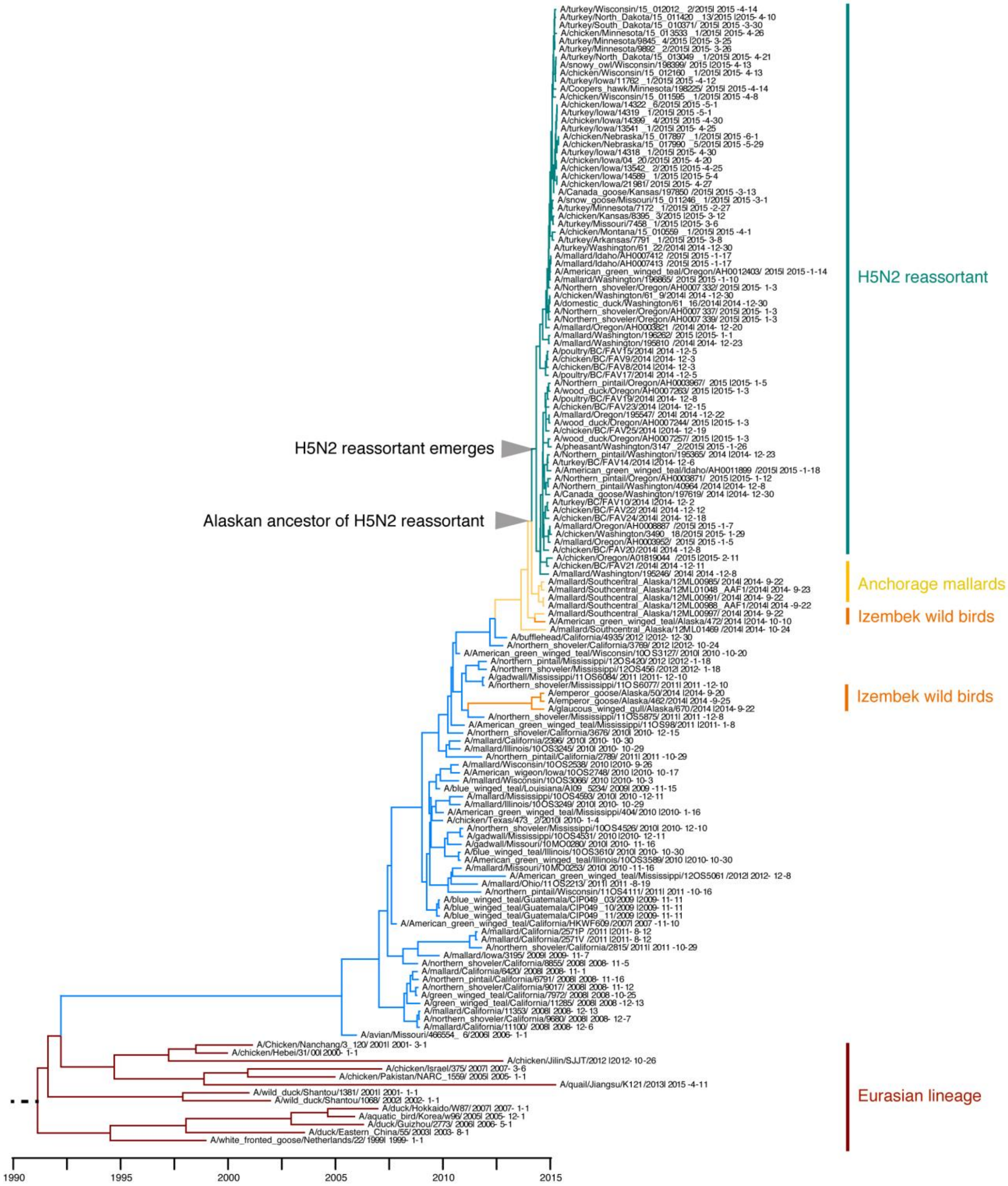

Technical Appendix Figure 9. Bayesian phylogenetic tree for the N2 segment constructed using sequences from clade 2.3.4.4 North American outbreaks. The tree has been time-calibrated using a relaxed molecular clock and branch lengths represent years. Nodes used to calculate time of most recent common ancestry (tMRCA: posterior probabilities >0.85) are marked with gray arrows.

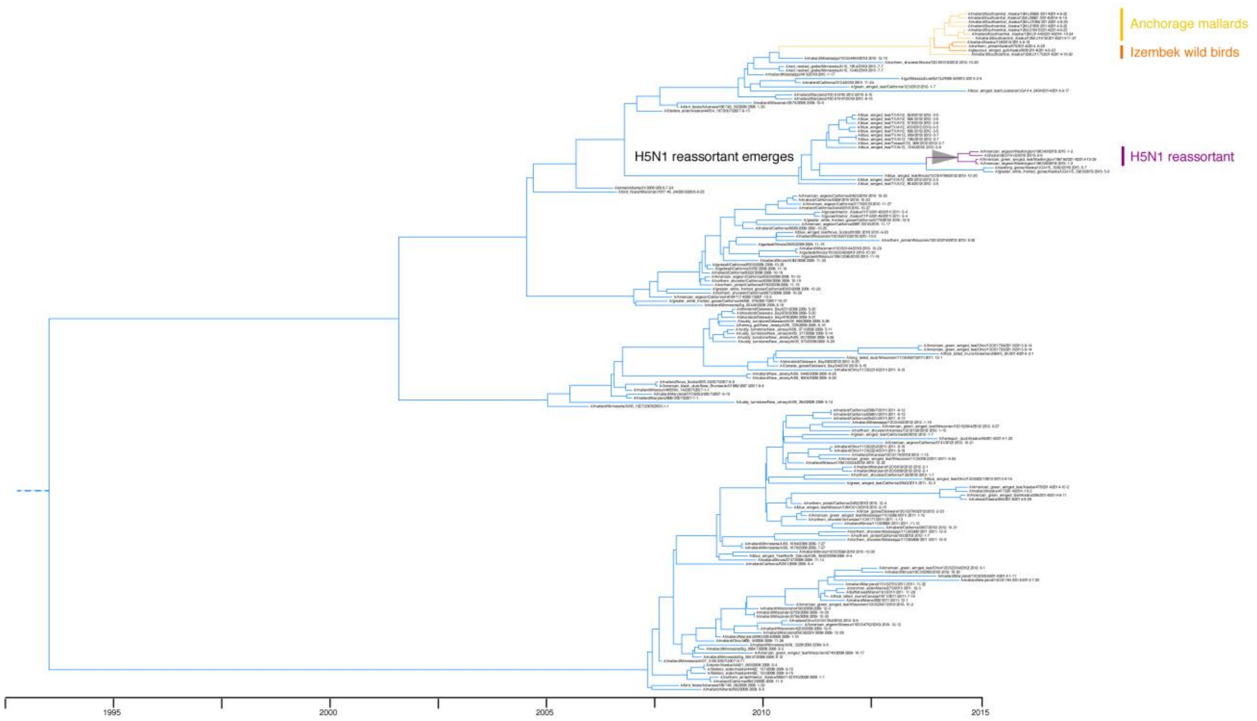

Technical Appendix Figure 10. Bayesian phylogenetic tree for the N1 segment constructed using sequences from clade 2.3.4.4 North American outbreaks. The tree has been time-calibrated using a relaxed molecular clock and branch lengths represent years. Nodes used to calculate time of most recent common ancestry (tMRCA: posterior probabilities >0.85) are marked with gray arrows.

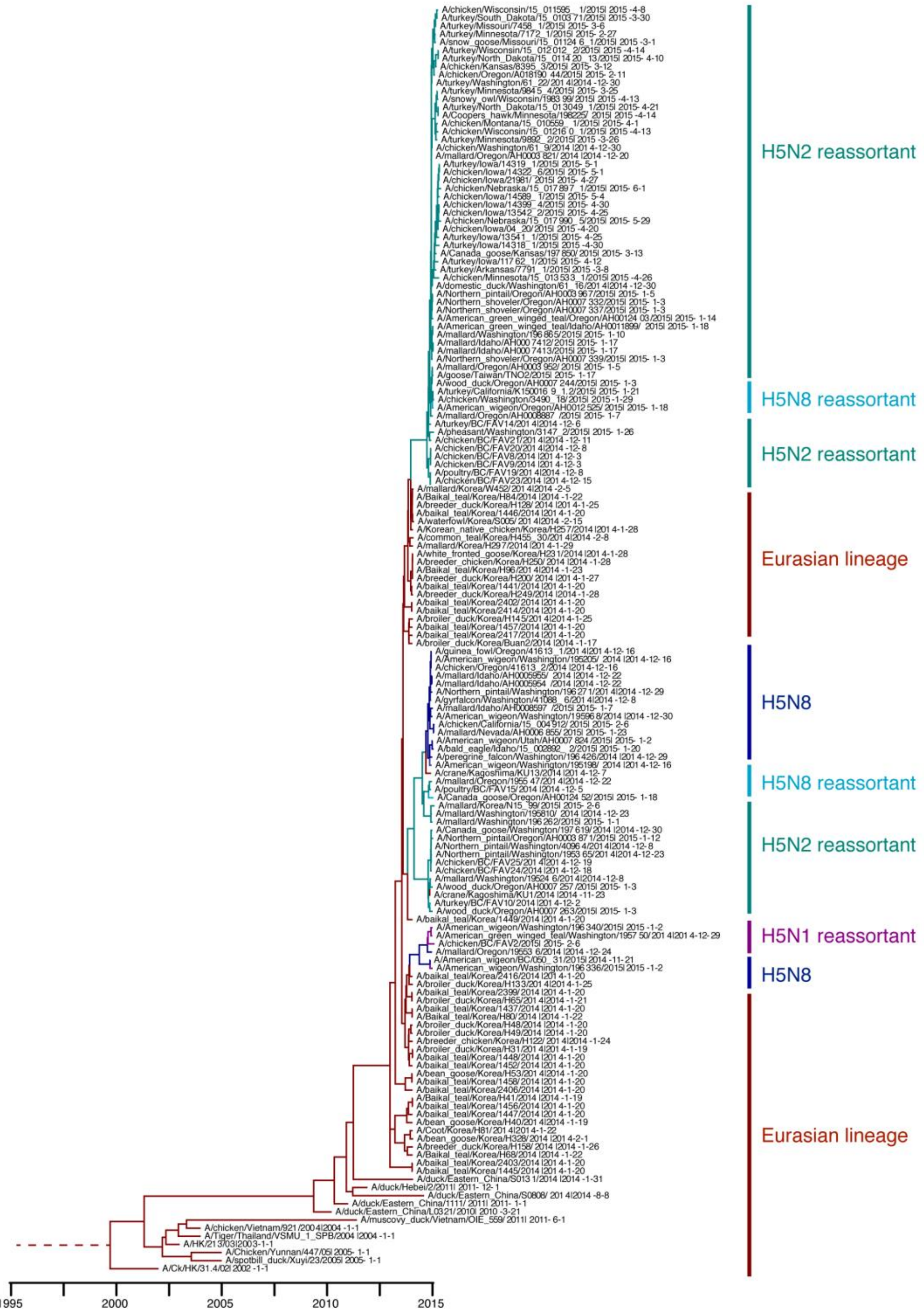

Technical Appendix Figure 11. Bayesian phylogenetic tree for the M segment constructed using sequences from clade 2.3.4.4 North American outbreaks. The tree has been time-calibrated using a relaxed molecular clock and branch lengths represent years. Nodes used to calculate time of most recent common ancestry (tMRCA: posterior probabilities >0.85) are marked with gray arrows.

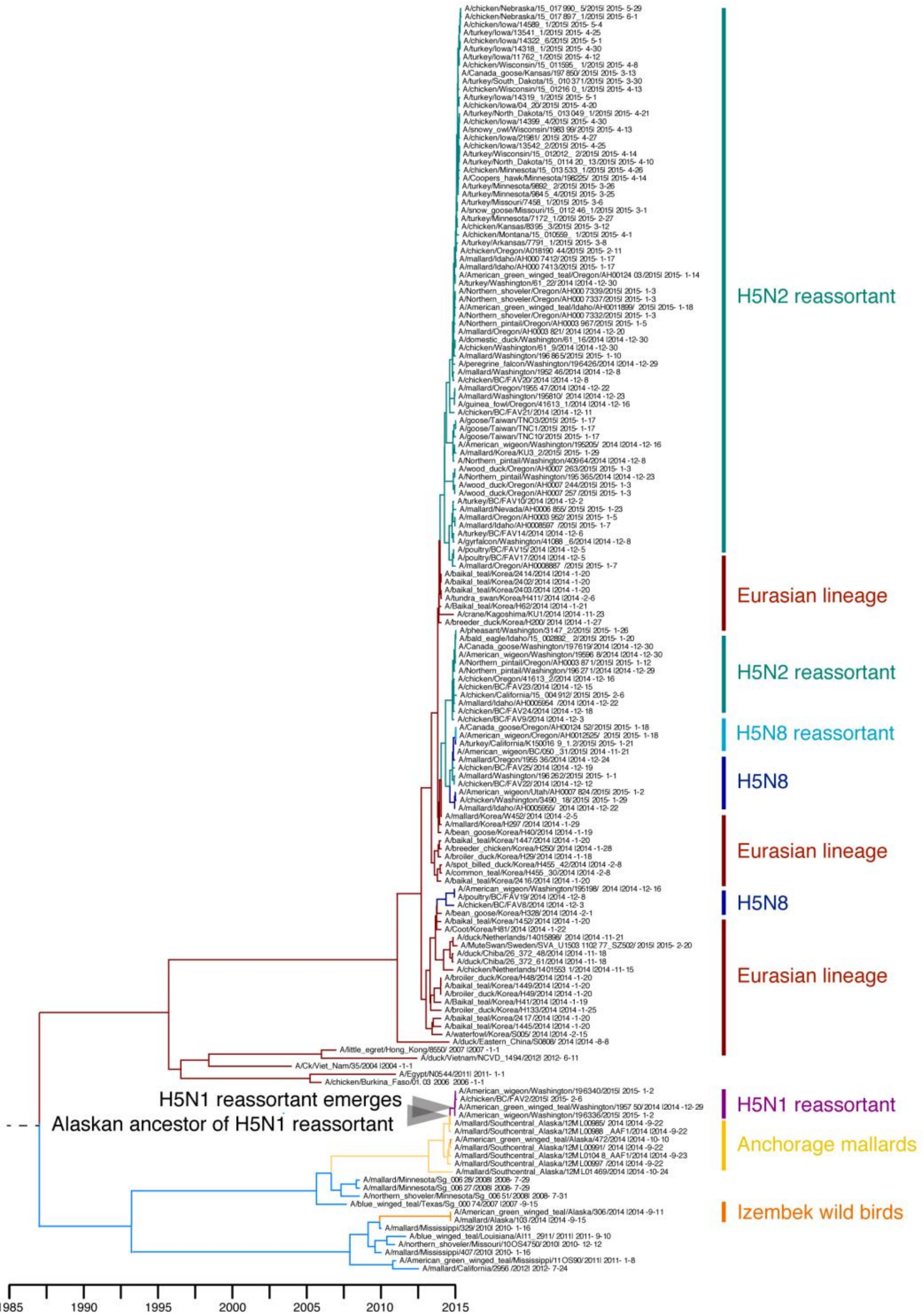

Technical Appendix Figure 12. Bayesian phylogenetic tree for the NS segment constructed using sequences from clade 2.3.4.4 North American outbreaks. The tree has been time-calibrated using a relaxed molecular clock and branch lengths represent years. Nodes used to calculate time of most recent common ancestry (tMRCA: posterior probabilities >0.85) are marked with gray arrows.

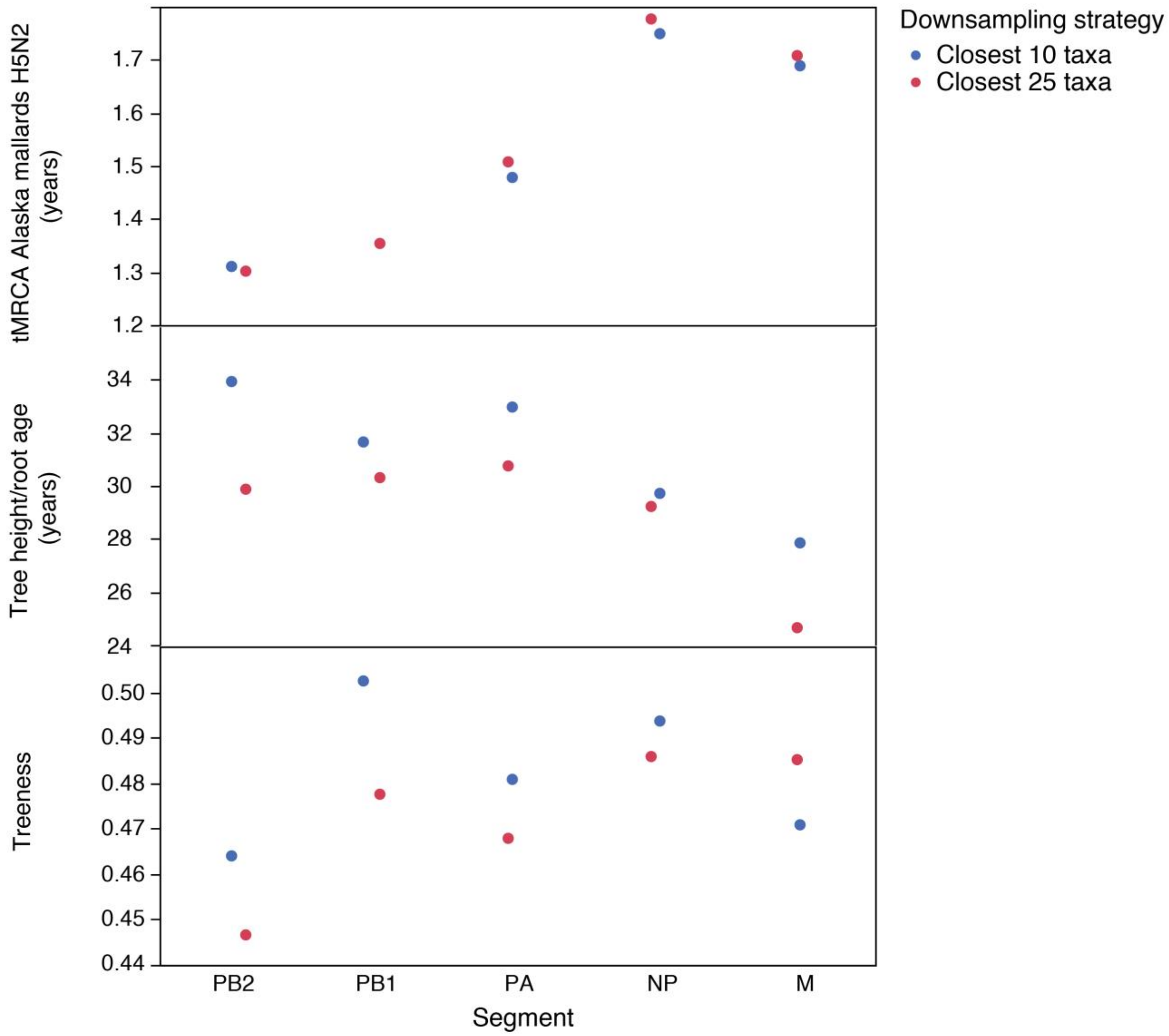

Technical Appendix Figure 13. The optimal down-sampling strategy for internal segments was determined using Bayesian tree statistics to compare the reliability of trees generated with the closest 10 or 25 relatives to the in-group (i.e., H5N8 clade 2.3.4.4 and Alaskan isolates from Anchorage and Izembek). The non-structural gene (NS) was not included due to allelic divergence.
